# Familial genetic risk for posttraumatic stress disorder: Associations with clinical features

**DOI:** 10.1002/jts.70053

**Published:** 2026-03-12

**Authors:** Ananda B. Amstadter, Linda Abrahamsson, James E. Hart, Jan Sundquist, Kenneth S. Kendler, Kristina Sundquist

**Affiliations:** 1 Virginia Institute for Psychiatric and Behavioral Genetics, Virginia Commonwealth University, Richmond, Virginia, USA; 2 Department of Psychiatry, Virginia Commonwealth University, Richmond, Virginia, USA; 3 Center for Primary Health Care Research, Department of Clinical Sciences, Lund University, Malmö, Sweden; 4 University Clinic Primary Care, Skåne University Hospital, Region Skåne, Sweden

## Abstract

In the present study, the novel *family genetic risk score* (FGRS) method, a reliable quantification of latent genetic risk, was applied to posttraumatic stress disorder (PTSD) to examine associations between genetic liability and clinical features of PTSD among 3,097,180 individuals in the Swedish national registries. FGRS was calculated based on lifetime PTSD status for first- through fifth-degree relatives and examined both in PTSD cases with any lifetime registration (PTSD total) and in cases with more than one registration (recurrent PTSD) in relation to sex, age at onset (AAO), recurrence, mode of ascertainment (inpatient [IP], outpatient specialty care [SC], primary care [PC]), and comorbidities. Sex differences were not found for recurrent PTSD, but for PTSD total, female registrants had a lower FGRS value compared to male registrants, *M* = −.017, 95% CI of difference [−.029−.005]. Higher FGRS was found at earlier AAO for PTSD total and recurrent PTSD, *p*s < .001, and scores were higher among individuals with comorbidities, *p*s < .001. Higher FGRS was related to the number of PTSD recurrences among both total PTSD and recurrent PTSD, *p*s < .001 (linear effect). For both PTSD types, FGRS scores were as follows: PC < SC < IP, *p*s < .001. The findings indicate that genetic risk for PTSD is associated with several clinical features of the disorder, which should be included in future studies of genetic risk for PTSD. Continued investigation of these clinical features in epidemiological and molecular genetic studies of PTSD is warranted to further validate the findings.

Posttraumatic stress disorder (PTSD) is a debilitating and prevalent condition. The lifetime prevalence of PTSD is estimated to be between 6.1% and 9.2% (de Vries & Olff, 2009; Goldstein et al., 2016; Koenen et al., 2017), and the lifetime prevalence for women is approximately double that of men (Kilpatrick et al., 2013; Tolin & Foa, 2006). Twin studies demonstrate moderate heritability for PTSD, with estimates ranging from 26% to 72% (Koenen et al., 2008; Sartor et al., 2011; True et al., 1993). In addition, meta-analytic results from a recent genome-wide association study (GWAS) by the Psychiatric Genomics Consortium (PGC) identified nearly 100 loci associated with PTSD case status, albeit with significantly lower observed heritability than twin and family studies (5.3%; Nievergelt et al., 2024).

Despite these recent advances in the genetic literature on PTSD, fewer genetic epidemiologic studies exist relative to studies on other disorders, leaving clinically important questions unanswered. To date, twin and molecular studies have focused on investigating the genetic risk for either the dichotomous diagnosis of PTSD presence or absence or for PTSD severity. Little is known about how genetic risk for PTSD may differ by clinical features, such as age at onset (AAO), recurrence, mode of ascertainment (e.g., diagnosis at an inpatient [IP], outpatient specialty care [SC], or primary care [PC] facility), or comorbidities. Clinical features, such as AAO, are important to study, as higher genetic risk has been found among cases with earlier AAO across a wide range of medical and psychiatric disorders (Kendler et al., 2023; Marenberg et al., 1994; Rissanen, 1979; Zdravkovic et al., 2002). If a higher genetic risk for PTSD is found at an earlier AAO, this would indicate that this feature is important to harmonize and study in molecular efforts to further aid in gene identification. Further, the current research is inconclusive regarding genetic sex effects, with a recent twin study finding support for sex differences in heritability (Amstadter et al., 2024), whereas the most recent molecular meta-analysis did not find sex differences (Nievergelt et al., 2024). Thus, alternative designs are needed to address these questions.

For this study, we examined these questions using a novel genetic epidemiologic tool— the *familial genetic risk score* (FGRS). The FGRS design is a validated measure of quantified genetic risk, correcting for familial environmental effects, and is derived from first- through fifth-degree relatives’ disorder history, as assessed using the extensive relevant Swedish registries (Kendler et al., 2021b). Like polygenic risk scores (PRS; Purcell et al., 2009), which utilize summary GWAS data and a matrix of an individual’s genotypes to calculate one’s genetic risk for a trait, the FGRS serves as an index of an individual’s genetic risk for a trait. This method, however, leverages the massive sample size and depth of generational phenotypic information within population-based registries to calculate a latent genetic risk score based on familial history. The FGRS is broadly calculated by averaging the weighted liabilities for each of an individual’s relatives, determined by time-at-risk, sex, birth cohort, and cohabitation effects, multiplied by their genetic relatedness to the individual being studied (i.e., proband), with corrections for birth year and county. The FGRS has the methodological benefits of a unified sampling frame and the elimination of response bias.

Prior FGRS analyses have demonstrated risk associations with various conditions, such as bipolar disorder, schizophrenia, and alcohol use disorder, and clinical characteristics including AAO, recurrence, and mode of ascertainment (Kendler et al., 2023, 2024). Here, we applied the FGRS design to PTSD for the first time, using data from Swedish national registries. We calculated FGRS for PTSD across all cases of PTSD, as well as those with recurrent episodes, to test for sex effects and examine associations between FGRS and clinical features of psychiatric conditions: AAO, recurrence, mode of ascertainment, and comorbidities.

## METHOD

### Participants

This study made use of nationwide Swedish registers (see Supplementary Material), linked to each other by each person’s unique identification number, which Statistics Sweden replaced with serial numbers for anonymity. We selected all individuals born in Sweden between 1960 and 1995 who had Swedish-born parents. The selection based on country of birth was made to ensure the availability of information from the registers. A wide range of birth years was selected to enable us to study AAOs for PTSD, as the diagnosis is more commonly registered with increasing calendar time, and allow for a long enough follow-up time after PTSD onset to examine recurrence. Individuals were followed until death, emigration, or study end (December 31, 2018). To ensure a reasonable chance of PTSD diagnosis, individuals were excluded if their follow-up time did not exceed either the beginning of 1995 or 10 years of age. Of note, Sweden implemented the use of the *International Statistical Classification of Diseases* (9th ed.; *ICD-9*; World Health Organization [WHO], 1978) in 1987. These criteria resulted in a cohort size of 3,097,180.

### Procedure

For each individual, we searched within the hospital discharge, outpatient care, and primary care registers for diagnoses of PTSD by searching for *ICD-9* and *ICD-10* (WHO, 2016) codes of 308 and F43.1, F43.0, and F62.0, respectively. Two different definitions of PTSD cases were applied: PTSD total (*n* = 189,161), operationalized as individuals with at least one PTSD registration, and recurrent PTSD (*n* = 45,203), operationalized as individuals having at least two PTSD episodes. An episode was defined as a registration without any previous registration within the last 90 days, as outlined in Kendler et al. (2025) and Rhee et al. (2025). We also examined two alternative definitions using 45 and 180 days to define an episode. Ethical approval was obtained from the Regional Ethical Review Board in Lund (No. 2008/409 and later amendments). All procedures contributing to this work complied with the ethical standards of the relevant national and institutional committees on human experimentation and with the Helsinki Declaration of 1975, as revised in 2008.

### Measures

We defined AAO as the age at first detected PTSD case registration. The number of PTSD episodes was used to evaluate PTSD recurrence. Mode of ascertainment was defined in hierarchical categories as IP, SC, or PC. All individuals who ever had an IP registration were defined as IP cases, whereas individuals with SC registrations but no IP registrations were defined as SC cases, and those with PC registrations only were defined as PC cases. Two forms of comorbidities were examined, defined as PTSD with substance use disorder (SUD) only, combining diagnoses of alcohol use disorder (AUD) and drug use disorder (DUD); PTSD with sleep disorder only; and PTSD with both SUD and sleep disorder. For definitions of AUD, DUD, and sleep disorder, see Supplementary Table S1.

### Data analysis

We calculated each individual’s FGRS for PTSD in accordance with the methodology developed by Kendler et al. (2021a), based on a mean of 32.2 first- through fifth-degree relatives (born 1932–1995) of the proband (see Supplementary Table S2). As with PRS, used in molecular genetic approaches, the FGRS aims to measure aggregate genetic risk, but the calculations are based on relatives’ phenotypic information as seen in registries rather than on genetic data in the form of measured DNA. Registry data also have the advantage of minimal selection bias. In a recent study that compared a similar registry-based approach to PRS, Dybdahl Krebs et al. (2026) concluded that for psychiatric disorders, both genetic instruments likely measure the same underlying additive genetic liability and should be seen as complementary. The FGRS method makes use of the Swedish Multi-Generational Register (see Supplementary Material) for building biological family trees. In this register, biological mothers and fathers were identified for 97% and 95%, respectively, of all Swedish-born individuals (Ekbom, 2011). The error rate of paternity has been estimated at a low of 1.7% (Dahlén et al., 2022).

For this study, the method began by constructing a dataset including all probands for whom the FGRS is going to be calculated. One row was created for each relative contributing to the proband. Information on the shared genetic effects between proband and relative (e.g., .50 for biological father, .25 for half sibling), year of birth and sex of the relative, age at PTSD registration, and age at follow-up (with December 31, 2018, as the latest possible date) was included. There are seven additional calculation steps for producing the FGRS. In Step 1, the morbid risk of PTSD of the relatives was calculated, taking into account the available follow-up time in each individual’s registry entries. Relatives with a PTSD registration were weighted with 1.0, and relatives without a PTSD registration were weighted with a low number, close to 0, if, for example, they had a long follow-up time. In Step 2, the binary variable for the relative’s PTSD registration was *z*-score transformed—one score for each sex and birth decade combination—to account for the varying rates of PTSD across groups. To correct the FGRS for shared environmental effects across all parent–offspring and sibling–sibling relationships (i.e., first-degree relatives), in Step 3, the most suitable sample for comparing the effects of genes plus environment to genes only was utilized to estimate the correction parameters. For parent–child relationships, we compared children who lived with their biological father for at least 13 years (categorized as “lived with father”) to children who never lived with or in the same community as their biological father (categorized as “not lived with father”). Logistic regression, with PTSD in offspring as the outcome, was applied, with independent variables of binary trait in father, type of father (lived with vs. not lived with), and their interaction. We used the interaction term as the difference in the effect between genes only and genes plus environment. For sibling–sibling relationships, we used a similar approach for estimation of parameters: a comparison of half-siblings who were reared together versus reared apart.

In Step 4, within each row in the data representing a unique combination of proband–relative, we calculated the product of the four factors: the proportion of shared genetic effect with the relative; the weight from Step 1, reflecting the relative’s available follow-up time; the *z*-score from Step 2, reflecting the relative’s adjusted sex and year of birth rates; and the correction parameter for cohabitation for first-degree relatives. In Step 5, within each proband, we calculated the FGRS as the average product across all relatives. In Step 6, we further corrected the FGRS for the number of available relatives via multiplication with a shrinkage factor (see Supplementary Table S2). Finally, in Step 7, the FGRS was standardized across probands (i.e., the 3,097,180 individuals in our study cohort) by year of birth and county of residence (i.e., the county where the proband lived for the longest time) into a *z* score (*M* = 0.0, *SD* = 1.0). We took this step because in registry data, there is a rise of registered diagnoses with calendar time, and there may be regional health care differences regarding the provision of diagnoses; the FGRS standardization is a simple way to remove some of these differences. For instance, it is reasonable to assume that one’s genetic liability for a disorder should not be affected by their year of birth. Additionally, this standardization allows for improved FGRS comparability with other disorders (Kendler et al., 2021a, 2021b, 2023).

We calculated lifetime PTSD prevalence across four different FGRS intervals (range: −1–3, due to the skewed appearance of the FGRS distribution), each with a length of 1.0 standard deviation. Hazard ratios (HRs) derived from Cox proportional hazards regression, corrected for year of birth and sex, and 95% confidence intervals (CIs) were calculated for the associations between FGRS and PTSD phenotype. We also tested whether point-biserial correlations between the two variables were significant. We performed linear regression models, one per independent variable, to examine whether FGRS profiles among individuals with PTSD (total and recurrent) differed regarding key clinical features; these models controlled for year of birth. The use of the FGRS as an outcome was chosen from a pragmatic standpoint to allow for comparability of the strength of associations between different clinical features and FGRS. The models should not be interpreted as though clinical features of PTSD have an effect on genetic risk, but rather that genetic liability for PTSD may have an effect on the clinical features of PTSD. In the linear regression models, sex was modeled as a binary variable, and AAO was modeled as a continuous variable on a 10-year scale and with linear and quadratic terms (cubic not significant); note that the scale is to aid in the interpretability of beta coefficients and has no impact on significance.

As a sensitivity analysis, individuals with birth years 1960–1969 were removed, rendering 134,819 PTSD total cases and 31,913 recurrent PTSD cases, as the AAO for individuals born during this period was inherently older because PTSD diagnoses were not available before Sweden began using the *ICD-9* in 1987. Number of PTSD episodes was modeled (based on the main and alternative definitions for cut-off values of 90, 45 and 180 days) among individuals with at least 5 years of follow-up available after PTSD onset (PTSD total: *n* = 70,216, recurrent PTSD: *n* = 23,586, applying the main cut-off-value of 90 days) as a discrete (i.e., numerical) variable with linear, quadratic, and cubic terms. Mode of ascertainment was included as a categorical variable, using PC as the reference. PTSD comorbidities were included as dummy variables, using PTSD only as the reference, compared to comorbid with sleep disorder only, comorbid with SUD only, or comorbid with both sleep disorder and SUD. Based on the linear regression models, at the mean year of birth for PTSD cases, we created figures by postestimating mean FGRS values, with 95% confidence intervals, for the different AAOs, number of episodes, modes of ascertainment, and PTSD comorbidities.

As a second analysis evaluating the impact of FGRS on recurrence, we conducted a Cox regression, measuring time from the first to second episode, with FGRS as the predictor and correcting for sex and year of birth. For evaluating the specificity of the FGRS to PTSD, we compared FGRS across individuals with PTSD only (PTSD total: *n* = 88,263 for PTSD, recurrent PTSD: *n* = 13,219) or major depression (MD) only (PTSD total: *n* = 435,570, recurrent PTSD: *n* = 504,484), applying a linear regression model with FGRS as the dependent variable and diagnosis type as the independent variable, correcting for year of birth. See Supplementary Table S1 for a definition of MD. The corresponding analyses were made to compare PTSD to both SUD and sleep disorder.

Data analysis was conducted from March 23, 2023, to October 21, 2025. Statistical analyses were performed using R (Version 4.4.2; Supplementary Table S3) and SAS (Version 9.4).

## RESULTS

As shown in Table 1, our cohort included 3,097,180 individuals (48.7% female), with an average age of 39.9 years (*SD* = 10.8) at the end of the follow-up period. Table 2 includes descriptive data for the sample, by PTSD definition. As expected, the prevalence of any lifetime registration for PTSD (i.e., PTSD total), as well as recurrent PTSD, in female registrants was nearly twice that of the prevalence in male registrants. The majority of the cases were detected only in the PC registries, followed in frequency by SC, then IP. Sex differences by location of registration were noted, such that for female cases, PTSD was more likely to be diagnosed in PC settings compared to male cases, whereas a higher proportion of male compared to female cases was detected in SC and IP settings. Considering comorbid diagnoses, the prevalence rates of MD, SUD, and sleep disorder were substantial, and they were higher among cases with recurrent PTSD.

Descriptively, the prevalence of both PTSD types increased at higher levels of FGRS such that the prevalence for PTSD total and recurrent PTSD were 5.1% and 1.1%, respectively, at FGRS values of −1.0–0.0, 7.2% and 1.8% at values of 0.0–1.0, 9.2% and 2.5% at values of 1.0–2.0, and 10.3% and 3.0% at values of 2.0–3.0. Notably, the prevalence of PTSD was 2–3 times as high among the highest versus the lowest of the compared FGRS interval levels. Among individuals with an FGRS greater than 3.0, the PTSD prevalence was even higher, at 12.3% for PTSD total and 3.8% for recurrent PTSD. Point biserial correlations between phenotype and FGRS were significant for both PTSD definitions, *p*s < .001. Using AAO for PTSD total and recurrent PTSD, Cox regression models demonstrated that the hazard ratios (*HR*s) for FGRS were 1.24, 95% CI [1.23, 1.24], and 1.30, 95% CI [1.30, 1.31], respectively.

There was no significant FGRS difference between sexes in cases of recurrent PTSD, *p* = .715 (linear regression); however, for PTSD total, female registrants had a significantly lower FGRS compared to male registrants, *p* = .004, although the effect size was small, *M* = −0.017, 95% CI of difference [−0.029, −0.005].

**FIGURE 1:**
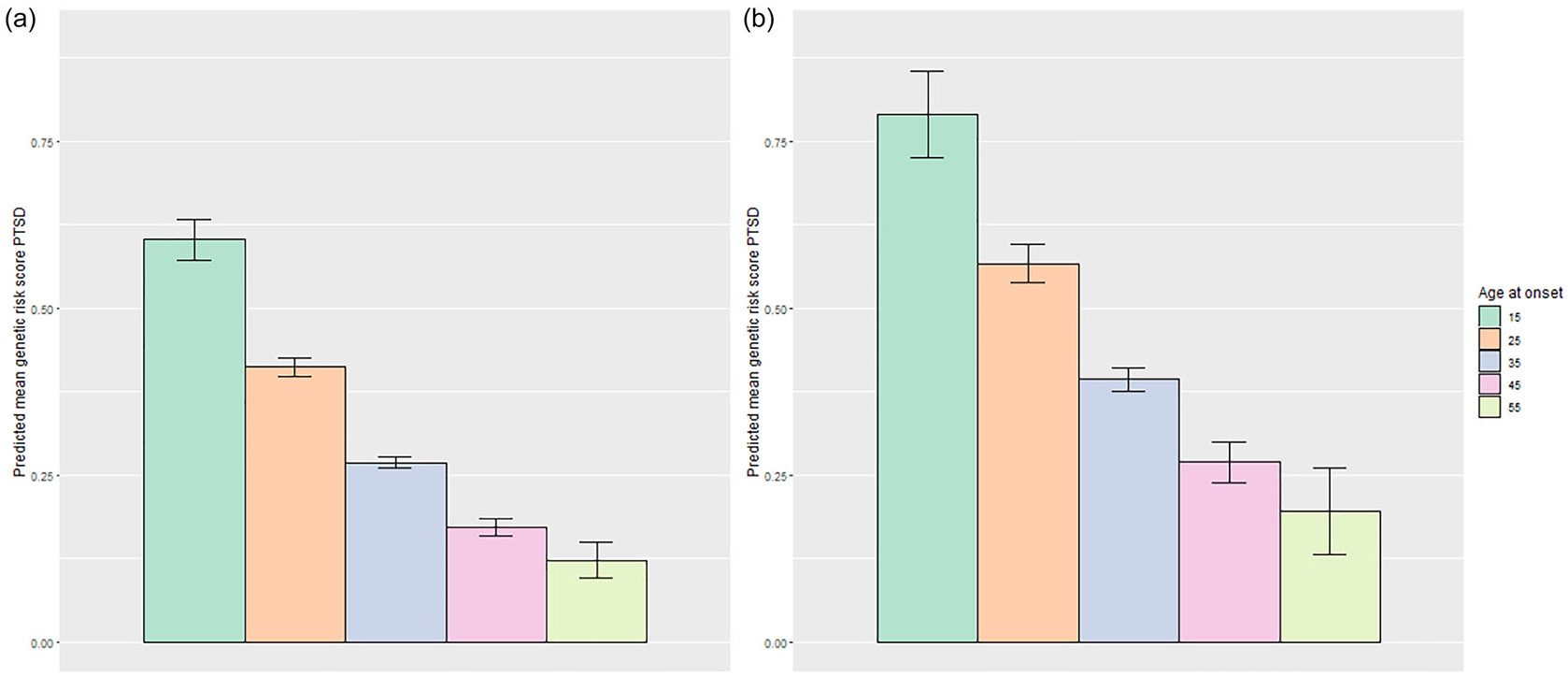
Predicted mean familial genetic risk score (FGRS) for (A) posttraumatic stress disorder (PTSD) total and (B) recurrent PTSD, by age at PTSD onset *Note:* The predicted mean standardized FGRS (*y* axis), with 95% confidence interval bars, for PTSD as a function of differences in PTSD age at onset (AAO). The figure depicts estimates from linear regression models at onset ages 15, 25, 35, 45, and 55 years for individuals with PTSD at the mean year of birth in the population for (A) PTSD total and (B) recurrent PTSD. The figure visualizes 98% of the range of AAO seen among the PTSD total cases, with wider confidence intervals in the tails of the AAO distributions.

Figure 1 depicts predicted mean FGRS by AAO, showing significantly higher FGRS at earlier age at first PTSD registration compared to later first registrations, meaning that individuals with high FGRS levels tended to be diagnosed at an earlier stage in life. We calculated FGRS change as a function of AAO for both PTSD definitions, on a 10-year scale, using linear regression analysis with linear and quadratic terms. For the linear terms, FGRS change was -0.2841, standard error of the mean (SEM) = 0.0201, *p* < .001, for PTSD total, and -0.3228, SEM = 0.0448, *p* < .001, for recurrent PTSD. For the quadratic terms, FGRS change was 0.0235, SEM = 0.0026, *p* < .001, for PTSD total and 0.0249, SEM = 0.0060, *p* < .001, for recurrent PTSD. In sensitivity analyses, excluding individuals born before 1970 and, thus, not eligible to have a young AAO given the timing of the diagnostic criteria implementation, the corresponding results for the linear and quadratic terms were similar to the main analyses, for PTSD total, linear: Δ*M*_FGRS_ = −0.2715, SEM = 0.0325, *p* < .001, quadratic: Δ*M*_FGRS_ = 0.0204, SEM = 0.0051, *p* < .001, and recurrent PTSD, linear: Δ*M*_FGRS_ = −0.3424, SEM = 0.0725, *p* < .001, quadratic: Δ*M*_FGRS_ = 0.0269, SEM = 0.0117, *p* < .001

**FIGURE 2:**
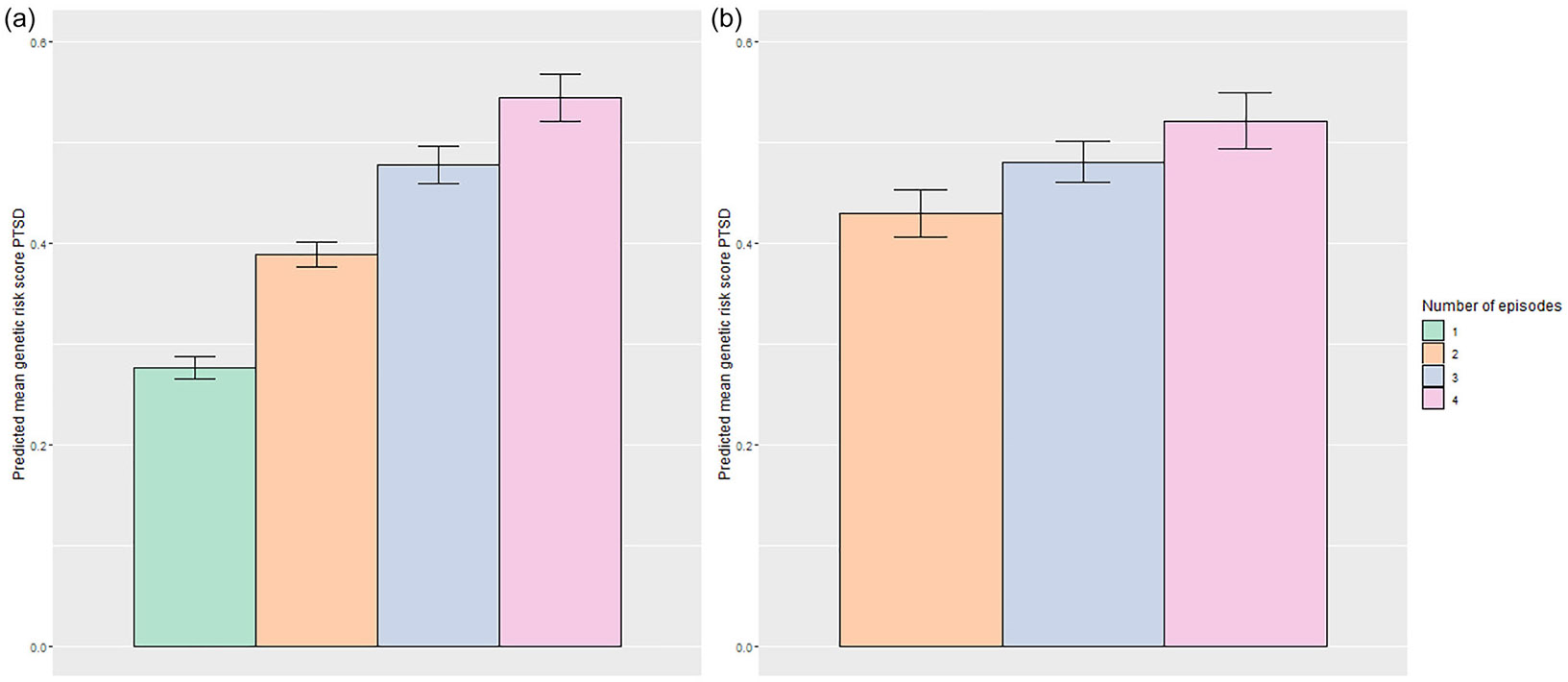
Predicted mean familial genetic risk score (FGRS) for (A) posttraumatic stress disorder (PTSD) total and (B) recurrent PTSD, by recurrence level *Note:* The predicted mean standardized FGRS (*y* axis), with 95% confidence interval bars, for PTSD as a function of differences in the number of PTSD episodes, as indexed by the number of independent registrations. The figure depicts estimates from linear regression models at 1–4 episodes for individuals with PTSD at the mean year of birth in the population for (A) PTSD total and (B) recurrent PTSD. The figure visualizes 94% of the range of the number of episodes seen among PTSD total cases.

FGRS was also associated with recurrence level (i.e., the number of independent registrations of PTSD), as depicted in Figure 2, meaning that individuals with high FGRS levels tended to have more independent PTSD registrations. Change in FGRS as a function of the number of episodes obtained from linear regression analyses showed mostly significant linear, quadratic, and cubic terms for both PTSD definitions. For the linear terms, FGRS change was 0.1505, SEM = 0.0106, *p* < .001, for PTSD total and 0.0768, SEM = 0.0208, *p* < .001, for recurrent PTSD. For the quadratic terms, FGRS change was −0.0136, SEM = 0.0016, *p* < .001, for PTSD total and −0.0057, SEM = 0.0026, *p* = .026, for recurrent PTSD. For the cubic terms, FGRS change was 0.0003, SEM = 0.0001, *p* < .001, for PTSD total and 0.0001, SEM = 0.0001, *p* = .175, for recurrent PTSD. When applying the alternative, less rigid, 45-day episode definition, FGRS change for the linear terms was 0.0902, SEM = 0.0061, *p* < .001 for PTSD total and 0.0448, SEM = 0.0101, *p* < .001, for recurrent PTSD. For the quadratic terms, FGRS change was −0.0054, SEM = 0.0006, *p* < .001, for PTSD total, and −0.0022, SEM = 0.0008, *p* = .007, for recurrent PTSD. For the cubic terms, FGRS change was 0.0001, SEM = 0.00001, *p* < .001, for PTSD total and 0.0000, SEM = 0.00002, *p* = .109, for recurrent PTSD. Applying the alternative, more rigid 180-day episode definition, FGRS change for the linear terms was 0.2551, SEM = 0.0245, *p* < .001, for PTSD total and 0.1465, SEM = 0.0666, *p* = .028, for recurrent PTSD. For the quadratic terms, FGRS change was −0.0364, SEM = 0.0065, *p* < .001, for PTSD total and −0.0167, SEM = 0.0131, *p* = .203, for recurrent PTSD. For the cubic terms, FGRS change was 0.0016, SEM = 0.0004, *p* < .001, for PTSD total and 0.0006, SEM = 0.0007, *p* = .389, for recurrent PTSD. In a second analysis evaluating the impact of FGRS on recurrence, when analyzing the time from the first episode to the second, using the main episode definition based on a 90-day cutoff, the hazard ratio for FGRS was estimated at 1.08, 95% CI [1.07, 1.09].

**FIGURE 3:**
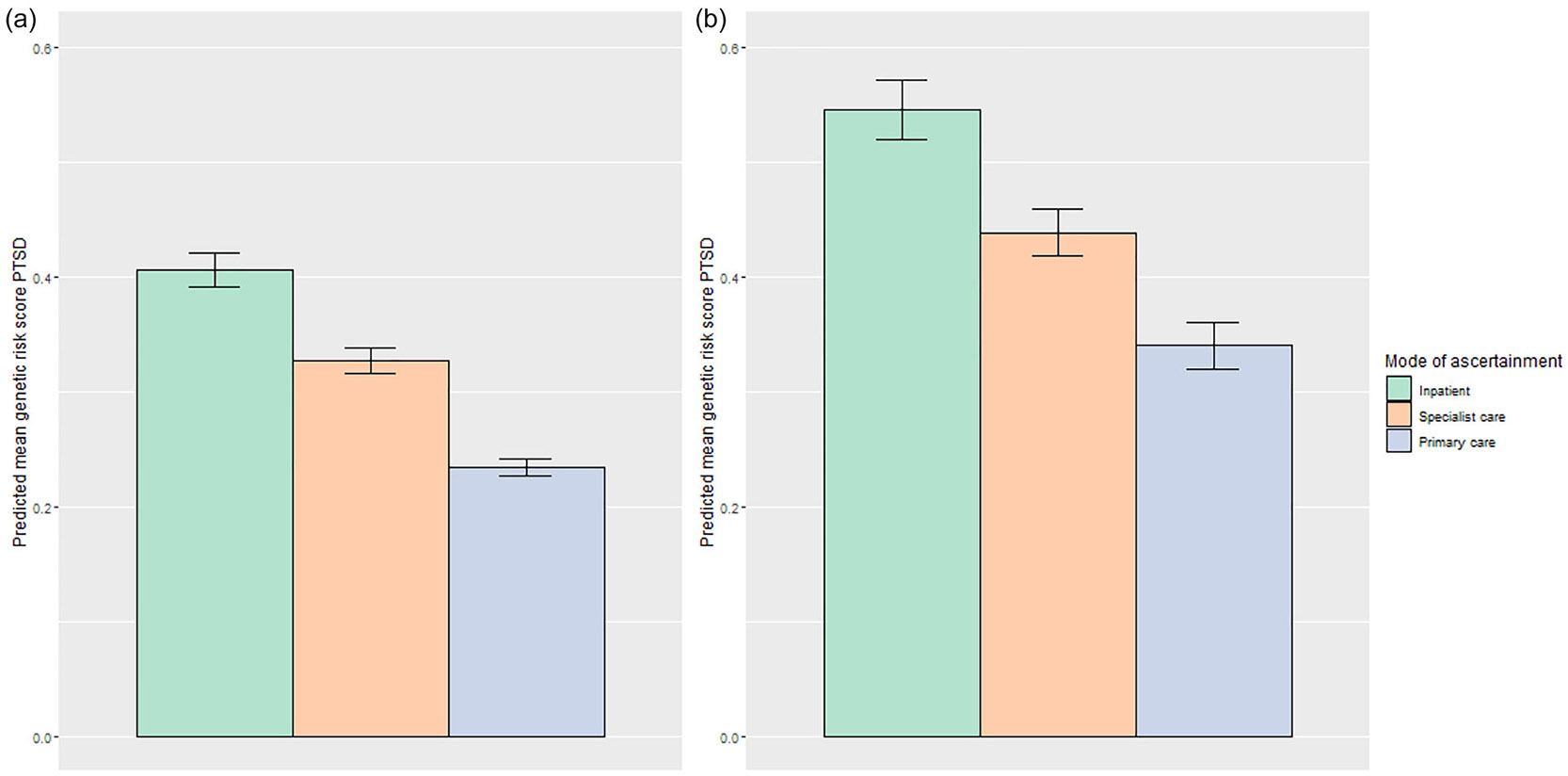
Predicted familial genetic risk score (FGRS) mean for (A) posttraumatic stress disorder (PTSD) total and (B) recurrent PTSD, by case identification source *Note:* The predicted mean standardized FGRS (*y* axis), with 95% confidence interval bars, for PTSD as a function of sources of ascertainment for PTSD, characterized as inpatient (IP), outpatient specialty care (SC), and primary care (PC) facilities. We used a hierarchy such that IP registration superseded other registrations, and SC clinic registration superseded PC clinic registration. The figure depicts estimates from linear regression models for individuals with PTSD at the mean year of birth in the population for (A) PTSD total and (B) recurrent PTSD.

Depicted in Figure 3, FGRS was highest for cases identified in IP settings, followed by SC, with the lowest in PC settings, meaning that individuals with a high genetic liability for PTSD tended to get a more severe form of the disorder, requiring a more complex level of care. For both PTSD definitions, FGRS change as a function of mode of ascertainment was obtained from linear regression analyses treating mode as a categorical variable, using PC as the reference level. For IP, FGRS change was 0.1720, SEM = 0.0086, *p* < .001, for PTSD total and 0.2056, SEM = 0.0168, *p* < .001, for recurrent PTSD. For SC, FGRS change was 0.0931, SEM = 0.0066, *p* < .001, for PTSD total and 0.0985, SEM = 0.0149, *p* < .001, for recurrent PTSD.

**FIGURE 4:**
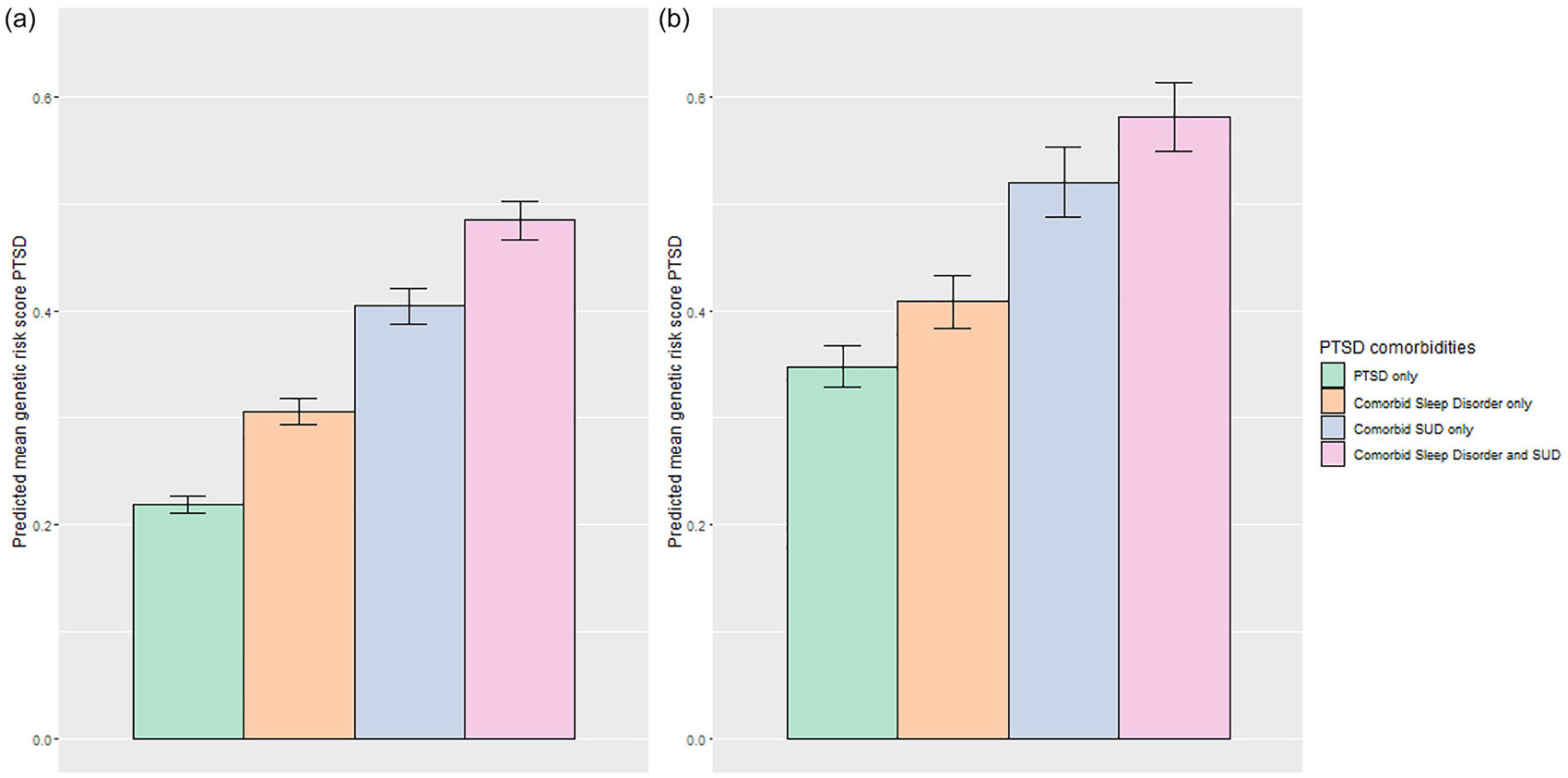
Predicted familial genetic risk score (FGRS) mean for (A) posttraumatic stress disorder (PTSD) total and (B) recurrent PTSD, by comorbidity status *Note:* Predicted mean standardized FGRS (*y* axis), with 95% confidence interval bars, for PTSD as a function of sleep disorder and substance use disorder (SUD) comorbidities. The figure depicts estimates from linear regression models for individuals with PTSD at the mean year of birth in the population for (A) PTSD total and (B) recurrent PTSD.

Depicted in Figure 4, FGRS was highest for cases that were comorbid with both SUD and sleep disorder, followed by those that were comorbid with SUD only, then with sleep disorder only, with the lowest FGRS for cases of PTSD without comorbidities. Change in FGRS as a function of comorbidities was obtained from linear regression analyses treating comorbidity type as a categorical variable, using PTSD only as the reference level, for both PTSD definitions. For cases that were comorbid with SUD only, FGRS change was 0.1852, SEM = 0.0093, *p* < .001, for PTSD total and 0.1724, SEM = 0.0193, *p* < .001, for recurrent PTSD. For cases comorbid with sleep disorder only, FGRS change was 0.0873, SEM = 0.0072, *p* < .001, for PTSD total and 0.0609, SEM = 0.0159, *p* < .001, for recurrent PTSD. For cases that were comorbid with both SUD and sleep disorder, FGRS change was 0.2654, SEM = 0.0102; *p* < .001, for PTSD total and 0.2337, SEM = 0.0190, *p* < .001, for recurrent PTSD.

When evaluating the specificity of the PTSD genetic risk score, the FGRS was found to be higher both for PTSD total only compared to MD only, with a mean FGRS change of 0.0815, SEM = 0.0042, *p* < .001, as well as for recurrent PTSD only compared to MD only, 0.1769, SEM = 0.0100, *p* < .001. For SUD, estimates were 0.0460, SEM = 0.0040, *p* < .001, for PTSD total and 0.1514, SEM = 0.0071, *p* < .001, for recurrent PTSD. For sleep disorder, we observed effect sizes of 0.1317, SEM = 0.0038, *p* < .001, for PTSD total and 0.2502, SEM = 0.0072, *p* < .001, for recurrent PTSD.

## DISCUSSION

We applied a relatively new method of capturing aggregate genetic risk, the FGRS, to study the clinical features of PTSD in a Swedish national sample. The FGRS design is a validated measure of latent genetic risk, which corrects for shared environmental effects, that is derived from an individual’s relatives’ history of PTSD as documented in the extensive relevant Swedish registries (Kendler et al., 2021b). FGRS, like a molecular PRS, can be conceptualized as an index of an individual’s genetic risk for a trait. In all analyses, we examine a broad definition of PTSD capturing any lifetime PTSD registration (i.e., PTSD total) as well as a more stringent definition of recurrent PTSD wherein two or more independent episodes were required; notably, the results are largely consistent between the two definitions, with a trend for higher effect sizes for recurrent versus total PTSD. Here, we discuss how we addressed five key questions that further the genetic epidemiologic knowledge on PTSD.

First, we found that the prevalence of PTSD was more than twice as high at the highest versus the lowest level of FGRS for both PTSD total and recurrent PTSD. Although prior studies have used complementary methods, such as PRS (Nievergelt et al., 2019) and examinations of PTSD risk in first-degree relatives (Leen-Feldner et al., 2013; Sack et al., 1995), research specifically leveraging the FGRS, which aggregates risk across all known biological relatives, is still nascent for PTSD. However, our finding of a higher prevalence of PTSD at higher levels of FGRS is consistent with prior FGRS work with registries focused on other disorders (Kendler et al., 2023, 2024), suggesting a high reliability of our findings.

Second, we did not find significant differences in FGRS by sex for recurrent PTSD; however, a significant sex difference was observed for PTSD total. Specifically, we observed a small effect size for PTSD total, such that male registrants had a higher FGRS compared to female registrants. As the prevalence of PTSD is higher in female individuals, this suggests that the genetic threshold for exhibiting the phenotype is lower in female individuals, whereas male individuals are more resistant to developing the disorder at the same level of genetic risk. However, we note that other sex differences (e.g., higher interpersonal violence exposure in female compared to male individuals, sex biases in diagnostic practices, differential perceived stigma by sex related to symptom reporting) could be unmeasured confounders. We also note that in our prior twin analyses, we found evidence of sex differences (i.e., higher heritability for female vs. male individuals and evidence of a qualitative sex effect; Amstadter et al., 2024). The most recent genome-wide association study (GWAS) meta-analysis on PTSD by the Psychiatric Genomics Consortium (PGC) reported no significant sex differences (Nievergelt et al., 2024). However, an earlier PTSD GWAS meta-analysis by PGC also indicated higher heritability in female compared to male individuals and identified some sex-specific loci (Nievergelt et al., 2019). There is a substantial literature on the question of sex differences expected in genetic risk under a multiple threshold model. For psychiatric disorders, often the model does not seem to apply, and sex differences in risk are lacking or quite small despite large prevalence differences in risk (e.g., Cloninger et al., 1978). Thus, additional work is needed to determine if there are genetic sex differences for PTSD.

Third, our finding that higher genetic risk for both definitions of PTSD (i.e., total and recurrent) was associated with earlier AAO is consistent with the broader literature linking increased genetic liability to earlier onset of psychiatric disorders, such as MD, using both FGRS (Kendler et al., 2021a) and PRS methods (Harder et al., 2022). Analyses from the PGC Depressive Disorder Working Group found that PRS for both bipolar disorder and schizophrenia were associated with earlier AAO for MD (Power et al., 2017), suggesting that higher genetic risk for various psychiatric disorders may be related to risk prediction of clinical features for other disorders. To our knowledge, this is the first demonstration of genetic risk predicting earlier AAO for PTSD. From a developmental psychopathology perspective, higher genetic risk for PTSD may impact both the likelihood of trauma exposure (i.e., gene–environment correlation; Kendler & Baker, 2007) as well as a heightened sensitivity to exposure (Koenen et al., 2009), both of which may help explain the association between FGRS and AAO. A key implication from this finding is that the incorporation of AAO into molecular studies could assist in the identification of cases with a higher genetic risk, thereby aiding in gene detection efforts. However, AAO may also index genetic heterogeneity (Kendler et al., 2024).

Our fourth main finding is that higher FGRS was associated with a larger number of registrations for PTSD, again for both PTSD definitions (i.e., total and recurrent). Prior FGRS studies within the registries have also demonstrated associations between higher FGRS and increased recurrence of other psychiatric disorders (e.g., bipolar disorder, MD, schizophrenia; Kendler et al., 2021a). This finding suggests that the FGRS may be a useful marker for identifying individuals who may be at risk for more severe or persistent courses of the disorder. Thus, future studies should examine incorporating the FGRS into risk prediction models to assist with treatment planning and resource allocation (e.g., the FGRS may be useful in identifying individuals who may benefit from early posttrauma intervention services or those who may need increased booster sessions following treatment to help prevent recurrence).

Fifth, we found an association between the FGRS and detection at higher levels of intensity of care (i.e., higher FGRS in IP settings, followed by outpatient SC, then PC), and the findings were consistent for both definitions of PTSD. This finding underscores that familial genetic risk may contribute to illness severity but also likely contributes to heterogeneity. It is possible that the detection of PTSD cases in higher-care settings is a secondary diagnosis, with a more severe disorder triggering higher levels of care. This possibility is consistent with our findings regarding higher levels of genetic risk for PTSD among cases with a comorbid diagnosis. This highlights the need for further subtyping efforts within psychiatry to better understand biologic risk and genetic heterogeneity.

To examine the specificity of the FGRS for PTSD, we compared FGRS levels among PTSD cases compared to cases of MD, SUD, or sleep disorder. In all analyses, the FGRS was higher among PTSD cases, suggesting that the FGRS for PTSD showed specificity and provides evidence of the construct validity of the FGRS PTSD instrument.

The study results should be interpreted in light of limitations. Diagnoses may be influenced by provider knowledge of familial history, which could influence both FGRS scores and case identification. Reliance on *ICD* codes does not allow for differentiation of the familial influences on trauma exposure versus their influence on PTSD. Studies using different methodologies (e.g., self-reported history of trauma exposure and PTSD symptoms) have found gene–environment correlations for trauma exposure itself as well as moderate heritability for PTSD (Lyons et al., 1993; Sartor et al., 2012; Stein et al., 2002). We previously estimated the degree of overlap of etiologic factors for self-reported trauma exposure with that of PTSD, finding that one fifth of the familial influences on PTSD overlap with those that influence trauma exposure (Amstadter et al., 2012). Thus, it is likely that the FGRS calculated for this study captures the risk for both trauma exposure and PTSD. Another limitation is that most PTSD cases were identified via nationwide PC data; thus, diagnoses were made by a PC physician rather than a psychologist or psychiatrist. We note that the reliability and accuracy of the PTSD diagnosis may be improved when it is identified repeatedly, or made in higher levels of care, which gives an alternative explanation for our findings that genetic liability is associated with certain clinical features. For reasons of comparability with FGRS for other disorders, as well as to enable enough power, the FGRS for PTSD had to be based on the definition of PTSD using the less stringent PTSD total operationalization rather than recurrent PTSD.

In general, the registry-based FGRS methodology is pragmatically performing well at large; however, corrections for cohabitation are not made in detail. We diminish the influence of parental and sibling diagnoses across all individuals the same amount, irrespective of whether a child was, for example, living with a parent for a few years or for the complete follow-up period. Unknown variables (e.g., if a child resides with each separated parent, if a child cohabitates with other relatives) preclude precise cohabitation correction. This means that there may be an over- or undercorrection for some individuals, but, on average, the FGRS will perform well. As there are linear trends in the increased frequency of psychiatric diagnoses, FGRS calculations correct for birth year. Additionally, our sample has generalizability limitations beyond native-born Swedes. Future studies utilizing other national registries should be conducted to determine if the findings replicate.

Taken together, these findings suggest that clinical features of PTSD, such as AAO, are important to include in future studies on genetic risk for the disorder. Our results are consistent with various research perspectives demonstrating that AAO, recurrence, mode of ascertainment, and comorbidity status may vary with genetic risk for psychiatric disorders, including MD, schizophrenia, drug use disorder, and attention deficit hyperactivity disorder (Harder et al., 2022; Kendler et al., 2023; Zhan et al., 2023). As highlighted in our results, the genetic risk for PTSD among those individuals with a diagnosis is not static but rather differs based on important sub-features of the disorder (e.g., AAO). These subphenotypes can inform molecular studies to examine how genetic risk operates. Indeed, work in depression has shown that the FGRS and PRS are, when correcting for their modest effect sizes, indices of the same underlying risk of disorder (Dybdahl Krebs et al., 2026). Thus, the present findings’ importance lies in the interaction between this crucial topic; the immense power of the detailed registry datasets, with a consistent sampling frame that eliminates response bias and decreases measurement error; and the ability to address these subtle and clinically important questions, which have not been able to be tested to date. These results have important implications for research and clinical care. Future research aimed at replicating the present findings across diverse samples and methodologies is needed.

## Supplementary Material

Online Supplement

Additional supporting information can be found online in the Supporting Information section at the end of this article.

## Figures and Tables

**TABLE 1:** Descriptive statistics for the full sample and for male and female registrants

	Total sample				Female				Male			
Variable	*M*	*SD*	*n*	%	*M*	*SD*	*n*	%	*M*	*SD*	*n*	%
Birth year	1977.33	10.52			1977.33	10.52			1977.33	10.51		
Age at follow-up (years)	39.87	10.83			39.84	10.90			39.90	10.76			
MD prevalence			536,468	17.3			337,333	22.4			199,135	12.5
SUD prevalence			239,066	7.7			73,950	4.9			165,116	10.4	
SD prevalence			353,188	11.4			190,776	12.7			162,412	10.2
FGRS PTSD	0.00	1.00			0.01	1.01			−0.01	0.99			

*Note:* MD = major depressive disorder; SD = sleep disorder; SUD = substance use disorder; FGRS = familial genetic risk score.

**TABLE 2:** Posttraumatic stress disorder (PTSD) and comorbidity data for the sample, by sex and PTSD definition

	Total sample				Female				Male			
	Total PTSD		Recurrent PTSD		Total PTSD		Recurrent PTSD		Total PTSD		Recurrent PTSD	
	*M*	*SD*	*M*	*SD*	*M*	*SD*	*M*	*SD*	*M*	*SD*	*M*	*SD*
FGRS PTSD in PTSD cases	0.28	1.26	0.43	1.37	0.28	1.26	0.42	1.38	0.30	1.26	0.43	1.35
Age at first PTSD diagnosis (years)	35.76	10.38	34.58	10.23	35.86	10.46	34.50	10.32	35.56	10.21	34.81	9.96
	*n*	%	*n*	%	*n*	%	*n*	%	*n*	%	*n*	%
PTSD prevalence	189,161	6.1	45,203	1.5	130,101	8.6	34,071	2.3	59,060	3.7	11,132	0.7
No. of individuals with ≥ 5 years follow-up	70,216		23,586		46,467		17,502		23,749		6,084	
PTSD episodes for individuals with ≥ 5 years follow-up^a^	1	1–2	2	2–4	1	1–2	2	2–4	1	1–2	2	2–3
Cases registered at IP care		14.2		23.9		12.1		21.8		18.9		30.4
Cases registered at SC care^b^		28.5		37.1		26.7		36.9		32.5		37.7
Cases registered only at PC		57.2		39.0		61.1		41.3		48.6		31.9
MD diagnosis		53.3		70.8		55.0		71.6		49.6		68.1
SD diagnosis		32.0		41.6		31.7		41.0		32.5		43.7
SUD diagnosis		20.9		30.4		15.9		25.7		31.7		45.0
MD and SUD diagnosis		15.2		25.0		12.5		21.9		21.0		34.4
SD and SUD diagnosis		9.3		15.5		7.3		13.0		13.6		22.9

*Note:* FGRS = familial genetic risk score; IP = inpatient; SC = outpatient specialty care; PC = primary care; MD = major depression; SD = sleep disorder. SUD = substance use disorder.

a Values are presented as median (*n* column) and interquartile range (% column).

b SC care but not IP care.
