# Supplementary material for: Familial genetic risk for posttraumatic stress disorder: Associations with clinical features
Source: J Trauma Stress. Author manuscript; Available in PMC 2026 Jul 27. (PMC13403086; doi:10.1002/jts.70053)
Supplement: Online Supplement [file NIHMS2193902-supplement-Online_Supplement.docx]

Supplementary Information

*Supplementary Methods (SM1)*

Ethical approval was obtained from the Regional Ethical Review Board in Lund (No. 2008/409 and later amendments). All procedures contributing to this work complied with the ethical standards of the relevant national and institutional committees on human experimentation and with the Helsinki Declaration of 1975, as revised in 2008. Participant consent was not required.

Total Population Register. The Total Population Register was created digitally in 1968 and includes yearly information, from the National Tax Board, on individuals registered in Sweden. It is possible to retrieve information on e.g. birth, death, immigration, emigration, migration within Sweden, place of residence, family information, civil status, etc. For more information, see [*https://www.scb.se/contentassets/8f66bcf5abc34d0b98afa4fcbfc0e060/rtb-bar-2016-eng.pdf*](https://www.scb.se/contentassets/8f66bcf5abc34d0b98afa4fcbfc0e060/rtb-bar-2016-eng.pdf)

Multi-Generation Register. The Multi-Generation Register is a register made up of persons who have been registered in Sweden at some time since 1961 and those who were born in 1932 or later. These are called index persons. The index persons, are listed together with their biological parents (in 2011, 97% of the Swedish-born index persons had listed biological mothers and 95% biological fathers, the error rate of biological paternity has been estimated to 1.7%). The information on biological parents is taken from the Swedish Tax Agency, Statistics Sweden and population registration from pastoral offices. In general, the mother giving birth to a child is registered as the biological mother. If she was married, the husband is registered as the father. If not married, but the mother and proposed father both agree on paternity, that father is registered. If there is no agreement, DNA tests may be used for determining the biological traits of the child. In 2024, more than 11 million index persons were included in the register. The Multi-Generation Register is a part of the register system for the Total Population Register. Every year, a new version of the register is created, including new index persons who immigrated or were born during the year. Information from the Multi-Generation Register may be disclosed for research and statistical purposes. For more information, see the following links and references:
*https://www.scb.se/vara-tjanster/bestall-data-och-statistik/register/flergenerationsregistret/
https://www.domstol.se/amnen/familj/foraldrar-och-barn/faststallande-av-foraldraskap/
Ekbom, A. The Swedish Multi-generation Register. In: Dillner, J. (eds) Methods in Biobanking. Methods in Molecular Biology, vol 675. Humana Press, Totowa, NJ (2011)
Dahlén, T., Zhao, J., Magnusson, P.K.E. et al. The frequency of misattributed paternity in Sweden is low and decreasing: A nationwide cohort study. J Intern Med 291, 95-100 (2022)*

National Patient Register. In the 1960's the National Board of Health and Welfare started to collect information regarding in-patients at public hospitals, the National Patient Register (NPR). Initially it contained information about all patients treated in psychiatric care and approximately 16 percent of patients in somatic care. The register at that time covered six of the 26 county councils in Sweden. In 1984, the Ministry of Health and Welfare together with the Federation of County Councils decided a mandatory participation for all county councils. From 1987, NPR includes all in-patient care in Sweden. Since 2001, the register also covers outpatient doctor visits including day surgery and psychiatric care from both private and public caregivers. For more information, see *https://www.socialstyrelsen.se/en/statistics-and-data/registers/national-patient-register/*

Primary Care Data. We also used information from our new Primary Care research dataset including individual-level information on clinical diagnoses from primary health care centers from the following Swedish counties: Blekinge (2009-2018), Dalarna (2005-2018), Gotland (2011-2018), Gävleborg (2010-2018), Halland (2007-2018), Jönköping (2008-2018), Kalmar (2007-2018), Kronoberg (2006-2018), Norrbotten (2001-2018), Skåne (1989-2018), Stockholm (2003-2018), Södermanland (1992-2018), Uppsala (2005-2018), Västra Götaland (2000-2018), Värmland (2005-2018), Västerbotten (1991-2018), Västernorrland (2008-2018), Västmanland (2014-2018), Östergötland (1990-2018), and Örebro (2006-2018). The retrieval of data differs due to timing of digitalization of patient records. In 2018, 99% of the Swedish population lived in these 20 counties. For more information see *Sundquist, J., Ohlsson, H., Sundquist, K. et al. Common adult psychiatric disorders in Swedish primary care where most mental health patients are treated. BMC Psychiatry 17, 235 (2017).*

The Population and Housing Censuses. Every fifth year between 1960 and 1990 Sweden conducted censuses. These registers include among other things, the population's employment, the composition of households and housing. For more information, see *https://www.scb.se/en/finding-statistics/statistics-by-subject-area/population-and-living-conditions/population-composition-and-development/population-and-housing-census-1960-1990-tpr/*

Prescribed Drug Register. The Swedish Prescribed Drug Register started in July 2005 and includes all prescribed drugs being fetched at pharmacies, linked to personal numbers. For more information, see *https://www.socialstyrelsen.se/en/statistics-and-data/registers/national-prescribed-drug-register/*

Cause of Death Register. The Cause of Death Register includes all deaths occurring in Sweden from 1961 (including for Swedish citizens dying abroad) and is updated yearly. There is also a historical register between the years 1952 to 1960. For more information, see *https://www.socialstyrelsen.se/statistik-och-data/register/dodsorsaksregistret/*

Criminal and Suspicion Register. The Swedish Criminal Register and the Swedish Suspicion Register includes individual-level information on all committed crimes from 1973 and all suspicions of crimes related to an individual from 1998. For more information, see *https://polisen.se/lagar-och-regler/behandling-av-personuppgifter/polisens-register/*

Supplementary Table 1 (S1)- Definition of disorders

| Disorder | Registers Used | Definition |
| --- | --- | --- |
| Posttraumatic Stress Disorder  (PTSD) | Hospital Discharge Register;  Outpatient Care Register;  Primary Care Data | Posttraumatic Stress Disorder (PTSD) was identified in the Swedish medical registries by ICD codes: ICD9: 308; ICD10: F43.1, F43.0 and F62.0. |
| Major Depression (MD) | Hospital Discharge Register;  Outpatient Care Register;  Primary Care Data | Major Depression (MD) was identified in the Swedish medical registries by ICD codes: ICD8: 296.0, 296.2, 298.0, 300.4; ICD9: 296B, 298A, 300E; ICD10: F32, F33. |
| Alcohol Use Disorder (AUD) | Hospital Discharge Register; Outpatient Care Register; Primary Care Data; Prescribed Drug Register; Cause of Death Register; Criminal Register; Suspicion Register | Alcohol Use Disorder (AUD) was identified in the Swedish medical and mortality registries by ICD codes: ICD8: 571.0, 291, 303, 980; ICD9: V79B, 305A, 357F, 571A-D, 425F, 535D, 291, 303, 980; ICD 10: E244, G312, G621, G721, I426, K292, K70, K852, K860, O354, T51, F10.1-F10.9; in the Suspicion Register by codes 3005, 3201, which reflect crimes related to alcohol abuse; in the Crime Register by references to laws covering crimes related to alcohol abuse (law 1951:649, paragraphs 4 and 4A and law 1994:1009, chapter 20, paragraphs 4 and 5) (only those individuals with at least two alcohol-related crimes or suspicion of crimes from both Crime Register and Suspicion Register were included); in the Prescribed Drug Register by the drugs disulfiram (Anatomical Therapeutic Chemical (ATC) Classification System N07BB01), acamprosate (N07BB03), and naltrexone (N07BB04). |
| Drug Use Disorder (DUD) | Hospital Discharge Register; Outpatient Care Register; Primary Care Data; Prescribed Drug Register; Cause of Death Register; Criminal Register; Suspicion Register | Drug Use Disorder (DUD) was identified in the Swedish medical and mortality registries by ICD codes: ICD8: 304; ICD9: 292, 304, 305E, 305F, 305H; ICD10: F11-F16, F18-F19; in the Suspicion Register by codes 3070, 5010, 5011, and 5012, that reflect crimes related to DUD; and in the Crime Register by references to laws covering narcotics (law 1968:64, paragraph 1, point 6) and drug-related driving offences (law 1951:649, paragraph 4, subsection 2 and paragraph 4A, subsection 2). DUD was identified in individuals (excluding those suffering from cancer) in the Prescribed Drug Register who had retrieved (in average) more than four defined daily doses a day for 12 months from either of Hypnotics and Sedatives (Anatomical Therapeutic Chemical (ATC) Classification System N05C and N05BA), Methadone (N07BC) or Opioids (N02A). |
| Sleep Disorder | Hospital Discharge Register;  Outpatient Care Register;  Primary Care Data | Sleep Disorder was identified in the Swedish medical registries by ICD codes: ICD10: F51 and G47. |

Table 2 (S2) - Steps for the Calculation of the FGRS

| The dataset for the calculations includes:  Column1 = Identification number of the proband (Born 1960-1995)  Column2 = Identification number of the relative (1st to 5th degree relatives; born 1932-1995)  Column3 = Proportion of shared additive genetic effects (0.03125 to 0.50) with the proband  Column4 = Year of Birth of relative  Column5 = Sex of relative  Column6 = Age at registration for trait  Column7 = Age at end of follow-up (2018-12-31 or age at death, or age at emigration whichever came first) |
| --- |
| **Step 1:** Using all unique relatives with a registration for the disorder, we non-parametrically estimated the distribution of *Age at first registration*. The empirical distribution is used to obtain weights for relatives without a registration for the disorder, in order to account for the proportion of the time-at-risk period they had completed at the end of follow-up. For example, for relatives at age x at end of follow-up, the weight corresponds to the proportion of relatives registered for the trait that had been registered at age x. For relatives born prior to 1958 we subtracted age at the end of follow-up with the following formula: 1958 - Year of birth of relative. This modification was done in order to control for registration effects (i.e, most registers in Sweden start in 1973 suggesting that relatives from early birth cohorts do not have the possibility to be registered at younger ages). Note that all relatives with the disorder are weighted one. |
| **Step 2:** Transform the binary variable (trait yes/no) into a z-score based on the threshold for each trait. The underlying liability of the individual is not assessable. Instead we estimated the mean of the underlying liability to obtain sex and birth decade specific Z-scores for relatives with the trait registration and relatives without the trait. We generate n random numbers from a N(0, 1) distribution and estimate the mean for relatives registered with the disorder (i.e., mean of the observations above the threshold) and for relatives without a registration (i.e., mean of all observation below the threshold). The thresholds are calculated for each decade of birth and sex. |
| **Step 3**: Correct for cohabitation effects. To estimate the cohabitation effect (i.e. “shared environment”), we created a database with all individuals in the Swedish population born in Sweden 1955-1990. We also included the number of years, during ages 0-15, that individuals resided in the same household as their biological father. We thereby were able to define two kinds of families i) “not-lived-with” father families (offspring never resided for more than 1 year in the same household or in the same community as their biological father); ii) “lived-with” father (offspring resided a minimum of 13 year in the same household as their biological father. We performed a logistic regression model with the binary trait in offspring as outcome and the binary trait in father, type of father, and their interaction as predictors. We used the interaction term as the difference of effect between genes only and genes + environment. The same approach was performed for half-siblings where we compared those who were reared together versus reared apart. The estimated cohabitation effects were made use of broadly across all parent-child and sibling-sibling relationships. The following interaction terms were used in the calculations:   \|  \| Parent/Children \| Siblings \| \| --- \| --- \| --- \| \| PTSD \| 0.83 \| 0.81 \| |
| **Step 4:** Calculate the product for each relative using the four components:   1. Z-score (reflecting sex and year of birth adjusted rates) 2. Weight (reflecting the proportion of risk period they had completed) 3. Cohabitation effects 4. Proportion of shared genetic effects (0.0625 - 1) with the proband |
| **Step 5:** Average the product calculated in step 4 across all relatives to a proband |
| **Step 6**: Correct for the number of relatives. We multiplied the results from step 5 with a shrinkage factor. Shrinkage factor (SF): B/(B+A/C). It produces more shrinkage if B and C are small and A is large.   1. the variance of the z-score of the disorder across all relatives, 2. the variance in the mean z-score across all probands, 3. the weighted number of relatives for each proband (sum of Column 3 across each proband). |
| **Step 7:** Correct for difference by year of birth and county differences. There are 21 counties in Sweden. For each proband we used the county they had resided in during the maximum number of years (measured from 1969 and onwards) We standardized the risk score by year of birth and county of the proband into a z-score with mean 0 and SD 1. This was then used as the FGRS in the analyses. |

Supplementary Table 3 (S3) – Details on R-packages used in statistical analyses

1. Wickham H. ggplot2: Elegant Graphics for Data Analysis. New York, NY: Springer-Verlag; 2016.
2. Neuwirth E. RColorBrewer: ColorBrewer Palettes. R package. 2022.
3. Wickham H, Miller E., Smith D. haven: Import and Export 'SPSS', 'Stata' and 'SAS' Files. R package. 2023.
4. Wickham H, François R, Henry L, Müller K., Vaughan D. dplyr: A Grammar of Data Manipulation. R package. 2023.
5. Barrett T, Dowle M, Srinivasan A, Gorecki J, Chirico M, Hocking T. data.table: Extension of ‘data.frame’. R package. 2024.
6. Wickham H. stringr: Simple, Consistent Wrappers for Common String Operations. R package. 2023.
